# A Review on the Use of Membrane Technology Systems in Developing Countries

**DOI:** 10.3390/membranes12010030

**Published:** 2021-12-27

**Authors:** Nur Hidayati Othman, Nur Hashimah Alias, Nurul Syazana Fuzil, Fauziah Marpani, Munawar Zaman Shahruddin, Chun Ming Chew, Kam Meng David Ng, Woei Jye Lau, Ahmad Fauzi Ismail

**Affiliations:** 1 School of Chemical Engineering, College of Engineering, Universiti Teknologi MARA, Shah Alam 40450, Selangor, Malaysia; nurhashimah@uitm.edu.my (N.H.A.); syazanafuzil@gmail.com (N.S.F.); fauziah176@uitm.edu.my (F.M.); munawar_zaman@uitm.edu.my (M.Z.S.); 2 Taman Industri Meranti Perdana, Pusat Teknologi Sinar Meranti, Techkem Group, No. 6, Jalan IMP 1/3, Puchong 47120, Selangor, Malaysia; davidng@techkem.com.my; 3 Advanced Membrane Technology Research Centre (AMTEC), Universiti Teknologi Malaysia, Skudai 81310, Johor, Malaysia; lwoeijye@utm.my (W.J.L.); afauzi@utm.my (A.F.I.)

**Keywords:** clean water treatment, developing countries, membrane technology, challenges, cost analysis

## Abstract

Fulfilling the demand of clean potable water to the general public has long been a challenging task in most developing countries due to various reasons. Large-scale membrane water treatment systems have proven to be successful in many advanced countries in the past two decades. This paves the way for developing countries to study the feasibility and adopt the utilization of membrane technology in water treatment. There are still many challenges to overcome, particularly on the much higher capital and operational cost of membrane technology compared to the conventional water treatment system. This review aims to delve into the progress of membrane technology for water treatment systems, particularly in developing countries. It first concentrates on membrane classification and its application in water treatment, including membrane technology progress for large-scale water treatment systems. Then, the fouling issue and ways to mitigate the fouling will be discussed. The feasibility of membrane technologies in developing countries was then evaluated, followed by a discussion on the challenges and opportunities of the membrane technology implementation. Finally, the current trend of membrane research was highlighted to address future perspectives of the membrane technologies for clean water production.

## 1. Introduction

The demand to increase clean water supply due to the proliferation of global population has prompted significant attention from world leaders [1]. In most urbanized areas of the world, clean water supply is produced from large-scale water treatment plants and is channelled through multiple networks of piping systems as tap water to the consumers [2]. These water treatment plants utilize various unit operations to purify raw water into safe and clean water before distribution [3]. The conventional water treatment process has been the dominant method to produce clean water on a large scale throughout the decades, especially in developing countries [4]. Some of the main advantages of the conventional process are the low capital and operational costs for the water treatment systems [5]. The main objectives of the treatment process are to remove the suspended solids and harmful bacteria through disinfection from the raw water.

Developing countries are generally defined as poor or middle-income nations based on the average income per person [6]. The water infrastructures of these countries are usually less developed due to financial constraints. There are various challenges faced by developing countries all over the world in providing a clean water supply to the people. In the Africa continent, a quantitative assessment conducted in Tanzania concluded the temporal variability model in drinking water faecal contamination against climate changes over 20 months [7]. Researchers from India, the second most populated country in Asia, have developed a cost-effective chlorine disinfection method for potable water [8]. These examples have indicated the emphasis on ensuring bacteria-free safe drinking water treatment processes in developing countries. The lack of water infrastructure has motivated many researchers to explore innovative, cost-effective water treatment methods.

Another major socio-economic obstacle faced by developing countries is the significant population living in rural or remote areas [9]. Due to the vast distance of certain rural villages and the low-density population, it is not economically feasible to connect water and electricity supply from the nearest city to these isolated locations. A study has indicated that an unregulated, privately financed or self-supply groundwater supply system in rural villages of Bangladesh pose a high risk of untested water quality [10]. Another analysis conducted in rural Kenya highlighted the failure risk for groundwater supply sources sustainability [11]. All these studies share similarities, pointing to the challenges of water security issues in rural areas in developing countries.

One of the suggestions to alleviate the clean water shortages in rural villages is to install regulated decentralized water treatment systems to cater to a small community. An economic feasibility study conducted in various developing countries such as Egypt, Nepal and Tanzania has highlighted some interesting and significant findings [12]. All three investigation sites have shown no realistic chance of recovering the initial or capital investment for the rural water treatment systems. In another study conducted on rural water treatment systems in Zimbabwe, similar economic drawbacks were also observed. The lack of technical support and expertise was also highlighted as one of the significant challenges to ensure proper operation and maintenance of these facilities [13]. Although decentralized small-scale water treatment systems have been proposed to solve rural clean water supply, the financial and technical aspects remain the most significant obstacles. Conventional water treatment systems offer economies of scale in urban areas, but they might not be suitable for rural areas with a small population and community lacking in various fields.

There are many challenges faced in the operation of these conventional systems which have prompted the emergence of other alternatives systems. In recent years, membrane filtration systems have emerged as one of the most widely used alternatives for large-scale water treatment plants and decentralized small-scale systems [14]. It has its advantages and drawbacks, for instance, the higher overall cost that hinders its adoption in developing countries [15]. For the last few decades, the thriving global economies have propelled many developing countries towards middle-income nations, enabling higher expenditure allocated for water infrastructures. These current developments present challenges and opportunities for researchers, scientists, policymakers, investors and stakeholders to explore mutually beneficial solutions.

**Figure 1:**
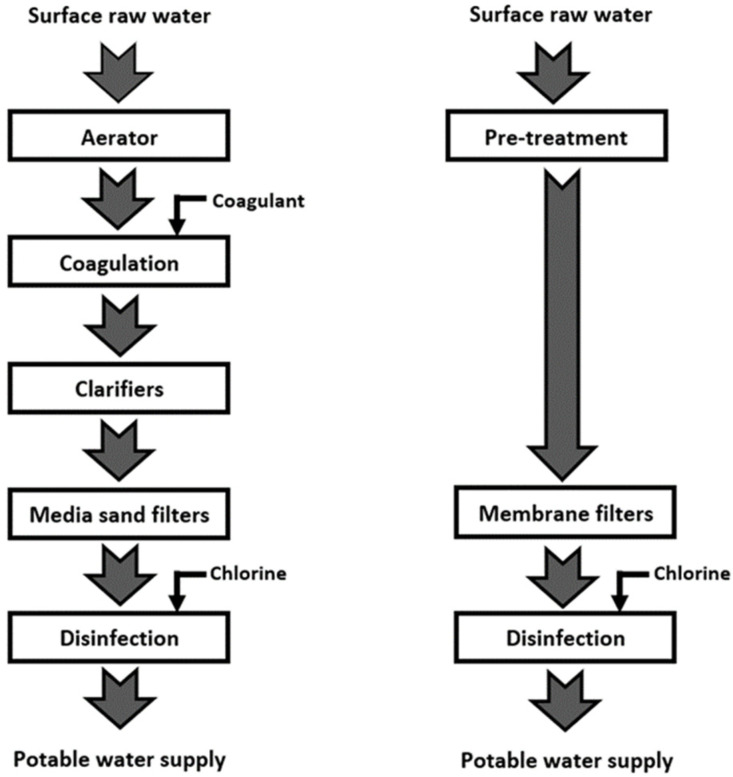
Block diagram comparison between conventional and membrane-based water treatment systems.

The industrial-scale conventional water treatment system is widely used in developing countries due to its significantly lower cost versus other high-end systems. Surface water is commonly the primary source of raw water to these conventional water treatment plants to supply clean or tap water to the public [16]. In the conventional system, inorganic based coagulant (such as aluminium sulphate and ferric chloride) is added into the raw surface water for the coagulation/flocculation process. Prior to the coagulant dosing, the raw water passes through a cascading aerator for the natural aeration process. The raw water with the coagulated particles and then passed through the clarification or sedimentation tanks for the solid–liquid separation. Final polishing is carried out with media sand filters before the chlorine disinfection process takes place. The chlorinated water is then ready to be supplied as tap water to the consumer. Figure 1 shows the summarized block diagram of the conventional water treatment system.

The main operational cost for the conventional system is on the purchasing of chemicals (coagulant, chlorine, etc.) as well as electricity. The production cost can go as low as USD 0.01/m^3^ of clean water produced in developing countries [5]. One of the most critical aspects of this system is the precise control of the coagulant dosage, pH and mixing to ensure the fine suspended solids are coagulated for the clarification and filtration processes [17]. Without a proper coagulation process, the system might fail to produce the desired quality of the treated water with turbidity below 5 NTU to comply with the World Health Organization (WHO) recommended drinking water standard. This is the major drawback of the conventional system, which relies heavily on the chemical coagulation/flocculation process to ensure an efficient solid–liquid separation in the system [18].

Conventional water treatment systems have been utilised extensively worldwide, including in high-income European countries [19]. In recent years, membrane-based filtration for large-scale water treatment systems has gained significant acceptance, particularly in developed countries. The membrane-based system’s higher capital and operational expenditure have caused the water production cost to be significantly higher for these countries. It has been suggested by the United Nations Development Programme (UNDP) that the water cost should not exceed 3% of household income [20]. In general, most household incomes in developed countries are significantly much higher than in developing countries, and thus higher water tariffs are imposed as well. The higher water tariff is necessary to sustain the membrane-based water treatment system, which usually provides a much more consistent and better water quality. Typically, the industrial-scale direct filtration membrane-based water treatment system consists of a pre-treatment process before the membrane filtration. After the filtration process, chlorine disinfection of the filtrate is carried out before supplying to consumers. Figure 1 shows the summarized block diagram of the membrane-based water treatment system.

The low footprint required and the consistent water quality produced by membrane systems have made it attractive, particularly in urban cities. The water tariff of these membrane systems can go as high as $ 1.52/m^3^ of water consumed [21]. The affordability of the consumers incurred a significant impact on the sustainability of these advanced systems. One of the most common membrane filtration systems for surface raw water treatment is ultrafiltration (UF) [22]. UF membranes with a pore size of 0.002–0.1 µm are commonly used to segregate fine solids from the filtrate. It is a low-pressure membrane filtration system that is suitable to replace conventional media sand filters. This low-pressure membrane filtration system with a much smaller footprint has made it attractive for large-scale applications [23]. As with other membrane filtration systems, the UF system comes with its own challenges and higher maintenance cost. Two of the most commonly used large-scale membrane water treatment systems are UF and reverse osmosis (RO) [24].

## 2. Membrane Technology for Water Treatment System

Typical municipal water treatment plant utilizing the conventional system heavily relies on the chemical-based coagulation/flocculation processes. The chemical dosage needs to be regulated periodically as they depend highly on the raw water source quality. Therefore, it is challenging to control the treated water’s quality without appropriate knowledge and monitoring techniques. In contrast to chemical-based treatments, membrane filtration systems produce microbiologically safe drinking water with no chemicals added, making it appropriate for drinking and sanitation requirements. The modular nature of membrane systems will allow various treatment capacities suited for the requirements.

### 2.1. Membrane Classifications

**Figure 2:**
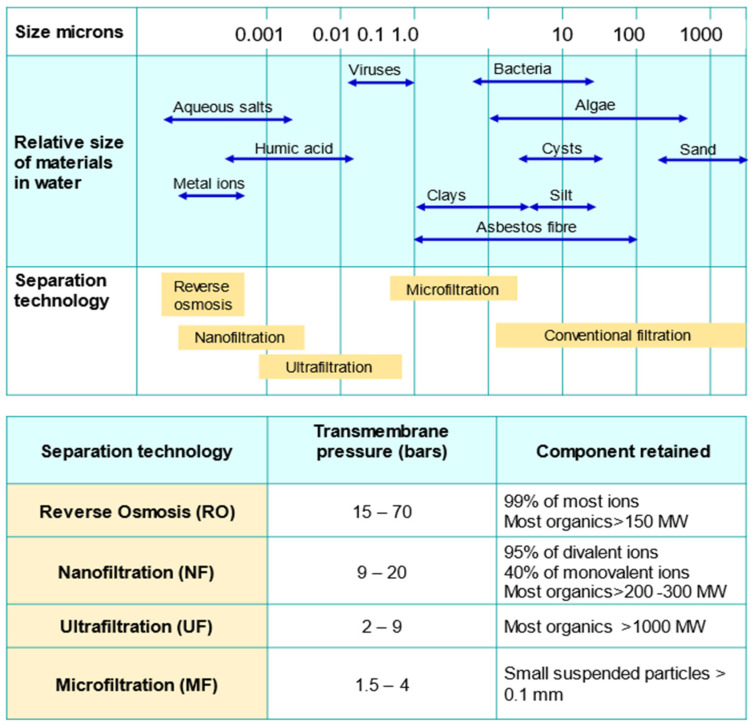
Membrane processes and application.

A membrane is a permeable or semi-permeable barrier that allows certain substances in the source waters to pass through the membrane while selectively restricting others. The separation of contaminants depends on the properties such as size and charge (Figure 2). The movement across the membrane requires a driving force that includes pressure difference, concentration gradient and a potential field to initiates ions movement. The pressure-driven membrane systems are categorized based on the operating pressure. Low-pressure membrane systems such as microfiltration (MF) and ultrafiltration (UF) are typically operated in the range between 10 to 30 psi. In contrast, high-pressure membrane systems such as nanofiltration (NF) and reverse osmosis (RO) require high operating pressures, varying between 75 to 250 psi. RO utilises a dense membrane where the pore size is less than 1 nm. Therefore, it is capable of removing almost all inorganic contaminants and the smallest organic molecules. In addition to being widely accepted as seawater desalination technology, RO has been observed to be efficient for the removal of synthetic organic contaminants (SOCs) such as herbicides and pesticides from polluted groundwaters.

Besides the pressure-driven membrane, an electrical potential can also be used to initiate dissolved ions to transfer across a water-impermeable membrane and has been regularly used for desalination purposes. This electrically driven membrane process is known as electrodialysis (ED) or reverse electrodialysis (RED). The induced direct current (DC) causes cation (+ve charged ions) to transfer across the cationic membrane to the cathode (-ve charged electrode) while restrained at the anionic membrane’s surface. Conversely, anions (-ve charge ions) move across the anionic membrane to the anode (+ve charged electrode) but are hindered at the cationic membrane. Consequently, a “dilute” stream-containing minimal salt concentration and a “concentrated” stream with higher salinity than the feed water can be obtained. The ion-exchange membranes (IEMs) used in this ED are typically expensive, contributing to the high cost of the membrane system.

The membrane is the “heart” of every membrane separation process as it plays a vital role in controlling the permeation of specific components. The selection of membrane process is subjected to the membrane properties. Membrane materials can substantially influence the properties and characteristics of the membrane, such as hydrophobicity and surface charge, thus, altering the separation characteristic of the membrane. Most of the membranes are fabricated from cellulose or non-cellulose organic polymers such as polyethersulfone (PES), polysulfone (PSF), polyvinyl difluoride (PVDF), and polypropylene. However, these polymeric membranes are not suitable for harsh conditions. As a result, inorganic ceramic membranes have been proposed to treat highly contaminated wastewaters such as oily water and highly turbidity water. The inorganic membrane is preferable to the polymeric organic membrane due to its excellent chemical stability, improved mechanical strength and can be used for high-temperature operation.

Membrane morphology can be categorized into symmetric or asymmetric structures. The symmetric structure is where the membrane has a uniform pore size or consistent morphology throughout the membrane’s cross-section. Conversely, an asymmetric structure comprises two main layers (a thin dense layer supported on a porous substrate) with diverse morphology and permeability properties. In asymmetric design, the pressure drop typically occurs in the thin dense separation layer, while the porous support layer aids in minimizing the transport resistance of the permeate across the membrane. The asymmetric membrane can be made either from one single material or a combination of different materials for separation and support layers.

Membrane performances are typically measured in terms of permeability rate or flux and selectivity. Membrane flux measures the diffusion’s rate of molecules across the membrane. It is the underlying step in membrane characterization and is highly reliant on the operating conditions of the membrane systems, such as pressure, temperature, and velocity at the membrane surface. Selectivity is typically based on membrane properties, particularly pore size or molecular weight cut-off (MWCO). MWCO signifies the lowest molecular weight solute (Daltons) in which 90% of the solute is retained by the membrane and commonly used to characterise UF. The significance of pore size towards membrane performance is constrained by the formation of a fouling layer on a membrane surface, which can assess rejection capability. This rejection characteristic is used to measure the performance of membranes.

Membranes for water treatment can be fabricated either in flat sheet or hollow fibre (capillary) form. A flat sheet membrane supported onto woven or non-woven support is typically assembled into a spiral-wound module for RO and NF systems. The spiral wound module is formed by wounding a membrane across a centre core tube for permeate, and a flow spacer material is then placed between the membrane. Hollow fibre membrane is typically utilized for drinking water MF and UF systems. The hollow-fibre membranes are bundled, and both ends are potted using resin before being housed in a cylindrical module.

### 2.2. Membrane Applications in Water Treatment

There are four types of raw water sources: groundwater, surface water, seawater, and rainwater that can be used for drinking and potable water supply. As these waters might contain various pollutants, appropriate treatment to remove disease-causing agents is required to ensure its suitability for drinking purposes. Groundwater is sourced from large underground aquifers, and a deep well must be drilled to take out the water. Surface water is water located above the ground, such as lakes, rivers, and streams. Groundwater commonly had minimal suspended solids, organics matters, and other potential foulants compared to surface water. Therefore, most of the membrane system configurations can be utilized for groundwater.

Surface water typically has higher suspended solids, dissolved organics, and microorganisms that require further treatment and filtration. In addition, the quality of surface water is directly affected by the use of land and human activities. In a more populated area, surface water quality might be low due to contamination from various sources. As the conventional surface water sources are drying up, seawater might be the best option as it is the most abundant source of aqueous solution in the world [25]. However, due to the high salinity of seawater, where the total dissolved solids (TDS) is around 35,000 ppm, it is vital to treat seawater and convert it into potable quality water with a TDS of between 200–500 ppm [26]. Rainwater is considered a high-quality source of water, but it might be acidic due to air contamination. Besides, rainwater could be exposed to zinc due to the rainwater collection system that is commonly collected through zinc roofing [27].

#### 2.2.1. Removal of Organic Compound

Natural organic matter (NOM) is a complex organic material discovered in groundwater and surface waters [28]. While it is not toxic, the presence of NOM can decrease the quality of the potable water by modifying its colour, odour and taste. It can function as a transporter of the poisonous organic and inorganic compounds such as pesticides and radionuclides in aqueous ecosystems [29]. Fulvic acids (FA, MW of 500–2000 Da) and hydrophobic humic acids (HA, MW ≤ 2000–5000 Da) are among the main components of NOM. These compounds may form strong complexes with heavy metals, causing the formation of organometallic complexes. Consequently, the transportation ability, bioavailability and toxicity will be increased, thus causing many health hazards [30]. As a result, it is essential to remove NOM during the water treatment processes.

Chlorination is utilized as one of the conventional disinfection treatments, but the interaction of chlorine with NOM forms a series of human carcinogenic constituents, including adsorbable organic halides (AOX), trihalomethanes (THM), halogenated acetic acids, halogenated aldehydes, ketones, halogenated acetonitryles, amines and other disinfection by-products (DBP) [31]. A membrane offers the possibility not only in removing DBP, but also NOM [32]. However, as the molecular size distribution of NOM is greatly varied from 1 nm to 0.45 μm, the removal efficiency depends on the properties of the membrane used [33]. NF and tight UF membranes were found to provide adequate initial disinfection (i.e., >4-log removal of all pathogens) and extensive removal of NOM. NF has been utilized in drinking water production in small communities (populations of 25–500) and able to remove pathogen and form potential DBPs precursors, which makes it suitable for NOM removal [34].

The elimination of NOM through RO also reduces the chlorine dosage needed for maintaining the residual chlorine concentration in the water distribution system due to biological activity reduction in the water [35]. This however is restricted by the high concentration of colloids and suspensions in the surface water [36]. UF and MF are capable of removing colloids and ionic and non-ionic organic compounds, which are suitable for NOM and high molecular weight DBP precursors removal [37]. However, an integrated system should be used mainly for medium- and low-molecular weight compounds [38]. For example, a UF module consists of a dense membrane (e.g., ca. 1000 Da) or a hybrid UF/MF system with coagulation, adsorption or oxidation processes have been proposed for the removal of humic substances from water [39,40,41,42].

Pharmaceutical active compounds (PhACs) and endocrine disrupting compounds (EDCs) are anthropogenic micropollutants frequently found in natural waters. EDCs can also enter the environment by industrial and municipal wastewater discharge. Activated carbon adsorption or advanced oxidation processes (AOPs) are typically utilised for this type of micropollutants [43]. However, when high content of NOM is observed in the feedwater, the technology proposed earlier is not attractive due to the increment of operating cost. Besides, by-products of undefined biological activity could be formed through AOPs. As a result, a pressure-driven membrane system seems to be more appropriate to be used. As EDCs are low MW pollutants, NF or integrated MF/NF systems can be employed where more than 90% removal rate of EDCs was observed [44]. Yoon et al. [45] showed that EDC removal using UF and NF membrane is based on hydrophobic adsorption and size exclusion mechanisms. A hybrid NF-AOPs system has been proposed for the removal of various PhACs from wastewater treatment plants. Complete removal of these pollutants was observed due to the synergistic effects of NF and AOPS [46].

Membrane bioreactors (MBRs) have also been investigated for removing pharmaceutical products and medicines from wastewater. It was observed that a longer retention time offered by MBR could improve the biological degradation and removal of PhAC and EDC. The production of plastics, typically PVC, has caused the presence of phthalates (a plastic agent) in the environment. A series of RO, NF and UF systems has been evaluated for phthalate removal in water, and the efficiency of removal was found to be in the range of between 97.6% to almost 99.9% [47]. Most of the contaminants discussed above can cause a direct impact on aquatic organisms even at a trace concentration, which has raised public concern, especially for water reuse purposes [48].

#### 2.2.2. Removal of Inorganic Compound

Desalination is a process of mineral components removal and is regularly used to obtain potable water from seawater (35,000 mg/L) and brackish underground waters (2000–5000 mg/L). RO has been seen as a competitive system to conventional distillation techniques. A pre-treatment system is required before RO to ensure that the permeate water meets the quality stipulated in the quality of drinking water regulation. The pre-treatment of raw water is vital to avoid membrane pollution such as fouling and scaling, extending the membrane’s life. A simpler membrane filtration system can be utilized for groundwater, as it is typically cleaner than surface water. The surface water needs to be treated extensively before it can be used, as it can contains various types and high concentrations of pollutants. Thus, a more complicated RO water treatment system including coagulation and adsorption might be required for surface waters. UF and MF have been seen as the most appropriate pre-treatment system prior to desalination. It removes suspended substances, some organic compounds and microbiological pollution, making it less contaminated feedwater for the RO system.

NF has been widely used as an alternative solution for water softening compared to chemical softening and ion-exchange methods due to its lower labour and operation costs. Hard water refers to a source of water with high mineral content, which can cause scaling [49]. The removal rate of hardness using NF is around 90%, and it is highly dependent on the type of membrane used and its operating conditions (water salinity and hardness). Recently, an integrated biological contact oxidation precipitation-UF-NF system was investigated, and it was observed that besides enhancement in removal rate, the life of the membrane could be prolonged [50].

Contamination of nitrate in water resources has become significant, and it can be from the discharge of industrial wastewater or nitrogen fertilizers used in agriculture. The maximum allowable concentration of nitrates in drinking water has been set up at around 50 mg NO_3_^−^/L as higher than that, will be toxic to humans [51]. RO has been used to treat borehole waters in rural areas in South Africa, and it was found to be effectively suitable for water denitrification where the nitrate-nitrogen was reduced from 42.5 mg/L to only 0.9 mg/L in the permeate [52]. In addition, the treatment cost of RO is comparable with the cost of ion exchange and electrodialysis systems.

Heavy metals pollutants such as iron, mercury, arsenic, chromium, copper, and lead might be present in the water source as a result of industrialization and urbanization. In many cases, a hybrid pressure-driven membrane and conventional water treatment systems is considered an attractive alternative towards environmental protection and the economy of the process. A hybrid pressure-driven combined air oxidation and MF system was used to remove iron from underground waters, especially when the amount of iron is high [53]. The system is more compact, and most importantly, high-quality clean water can be attained irrespective of the raw water quality. In order to reduce the amount of arsenic in drinking water, RO/UF along with hybrid coagulation-MF/UF can be utilized. RO TFC-ULP Koch membrane removes 99% of As (from 60 to 0.9 μg/L) from groundwater while DK2540F Desal membrane is able to remove 88–96% of As. NF membranes are also applied to As removal, and 97% removal of As (V) was obtained for membrane NF-70 (by FilmTec). The mechanism of arsenic removal using NF was found depending on the sieving separation and electrostatic repulsion between ions present in the treated solution and charged membrane.

### 2.3. Progress of Membrane Technology for Large-Scale Water Treatment Systems

The significant drop in surface raw water quality due to human activities has caused a lot of difficulties for the conventional water treatment system to produce the desired treated water quality [54]. As an alternative, membrane-based water filtration systems can offer a more robust and consistent solid–liquid separation process to produce good quality potable water. Membrane filtration has been utilized in commercial water treatment systems for decades. Its global market has reached up to an estimated USD 26.3 billion in the year 2017 and an expected 8.5% yearly growth [55]. Large-scale membrane water treatment systems are mostly commissioned in developed countries with high per capita income due to various reasons. First, the small footprints of membrane systems compared to the conventional system made it feasible to construct on land-scarce urban cities [56]. In addition, the modular type membrane system could be upgraded easily within a short time to suit the increase of water demand accordingly [57]. The quality of membrane has also improved significantly with distinctive advantages such as low fouling properties, higher chemical resistance tolerance and good mechanical strength, to name a few. Within the past few decades, commercial-scale membrane water treatment plants have significantly increased the total production of treated water.

The current shift of technology from conventional to membrane-based water treatment systems in developed countries is driven by the robustness of the latter. In developed countries, wide implementation of water treatment technology, including membrane filtration, has minimized the contamination risk of potable water. European countries such as Spain have upgraded their existing water treatment facilities in Barcelona with UF and RO systems to improve the water supply quality [58]. One of the push-and-pull factors for the proliferation of these membrane systems is the increasing demand for high water quality due to global population increase and rapid industrialization [59]. Water reclamation using membrane technology has been widely applied in Singapore to produce NEWater to cater for 40% of the country water demand [60]. In Singapore, a submerged hollow-fibre membrane was first installed in Chestnut Avenue Water Works (CAWW) in 2003 to produce drinking water. Then, the system was enhanced by adding submerged ceramic-based membrane system leading to a design capacity of 36,400 m^3^/d. This allow higher flux system to fulfil the demand [61]. In India, the desalination technology is essential for meeting the freshwater requirements. However due to high fossil fuel cost and remote area, it is expensive to establish large water treatment facilities. Therefore, the integration of renewable energy can helps to minimize the energy usage and cost [62]. All these positive developments are pointing towards the high feasibility of large-scale membrane water treatment systems.

### 2.4. Advantages and Disadvantages of Membrane Technologies for Water Treatment

One of the more prominent advantages of membrane filtration compared with the conventional water treatment system is the more consistent quality of the treated water [63]. Conventional systems rely heavily on the coagulation–flocculation process for the fine, suspended particles to form dense flocs. These flocs shall subsequently be separated in the clarifier and media filter through depth filtration. Failure to observe an effective coagulation–flocculation process will render poor solid–liquid separation in these units operation. Unlike the conventional system, membrane filtration (e.g., UF) is based on a cake filtration mechanism which enables effective solid–liquid separation even without the coagulation-flocculation process [64]. Consistent water quality is a critical aspect, especially in manufacturing, whereby it can significantly impact the end product outcome. Many membrane filtration systems are installed in these factory premises to ensure high control of the treated water consistency and quality for their production or manufacturing process. It has been reported that membranes are more effective in removing contaminants such as bacteria and dyes than adsorption technology [65]. All these findings have consolidated that, with a proper design of the membrane water filtration systems, high quality of filtrate can be produced.

Another distinctive feature of membrane-based water treatment systems is the relatively small footprint required compared to the conventional sand/media filtration system [5]. The modular concept of most pressurized membranes enables fast and easy upgrades to increase the treated water output capacity. Population growth of high-density urban cities are expected to be 1.9% from the year 2020 until 2030 [66]. This translates to roughly about 10% increase in water demand every five years for domestic usage in the urban areas. Under limited or scarce land and the urgency for municipal water treatment plant upgrades, a membrane-based system seems to be a feasible option to be considered.

Overall capital and operational cost of industrial-scale membrane water treatment systems are generally higher than the conventional freshwater system [5]. This is a significant drawback, especially for many developing countries whereby the per capita income of the population is much lower than the global average. Besides the higher overall cost incurred, one of the most prominent disadvantages of membrane systems is the fouling issue [67]. Membrane fouling issues have been widely reported in many industrial-scale membrane water treatment plants [68,69,70]. Membrane fouling causes higher operational cost due to increase trans-membrane pressure to maintain the desired filtration flux. In order to mitigate membrane fouling, chemical cleaning and periodic monitoring of the system are often carried out.

Membrane filtration systems, especially UF and MF, automatically require backwashes sequence as frequent as 2 to 4 times per hour, depending on feed water quality and flux [71]. This is essential to ensure minimal fouling before the next sequence of filtration begins. It is common for large-scale membrane water treatment plants to be equipped with computerized process control hardware and software to ensure uninterrupted operation with minimal human intervention [72]. All these facilities require periodic maintenance, which necessitates additional costs to the system operators [73]. Although production cost of the membrane has dropped due to economy of scale, another relevant expenditure such as electricity, labour, spare parts cost has increased due to inflation. The issue of affordability remains one of the biggest challenges, especially to most developing countries towards the adoption of large-scale membrane water treatment systems. 

### 2.5. Fouling in Membrane Systems

**Figure 3:**
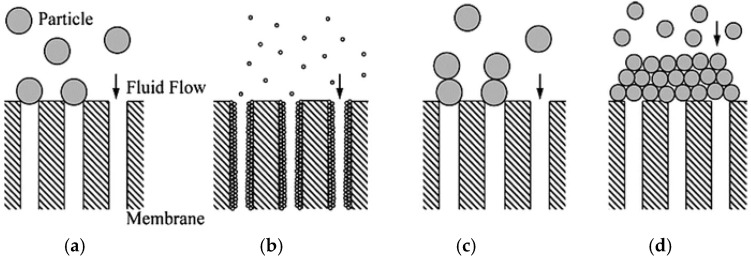
Illustration on membrane blockage by retained molecule: (**a**) complete blocking, (**b**) standard blocking, (**c**) intermediate blocking, and (**d**) cake filtration. Reprinted from [76] with permission from Taylor & Francis Ltd. 2013.

Membrane systems need to be adequately maintained to prolong their life cycle. The main problems faced by the membrane system are fouling and scaling. Fouling, including cake layer formation or pore blocking by organics is the result of concentration polarization (CP). CP is a phenomenon that take places when there is an increase of rejected component at the boundary layer near to the membrane surface [74]. This can cause damage to the membrane, leading to the decrease of permeate flux and product water quality. In contrast, membrane scaling occurs when dissolved substances precipitate from the solution and accumulate on the membrane surface or lodge in its pores. Organic molecules with a bigger size than the membrane’s pores can cause adsorption on the surface, which can cause blockage at the membrane entrance. This blockage forms a cake layer after a period when the membrane system is put into operation. This will reduce the cross-sectional area of the membrane and cause resistance in the membrane process. Consequently, a reduction of flux could occur. Figure 3 shows an illustration of blockage in the membrane system. Four models are used to describe the blocking phenomena, including complete blocking, standard blocking, intermediate blocking and cake filtration. Complete blocking assumes that all the molecules that reach the membrane surface completely block the membrane pores’ entrance. The standard blocking occurs when the molecules enter through the membrane’s pores and deposit over the pore walls [75]. Therefore, the volume of membrane pores is assumed to decrease proportionally with permeate volume. As for cake filtration, the molecules are considered bigger than the membrane pores and thus only deposit on the membrane surface. The intermediate blocking is typically less restrictive and occurs due to the simultaneous pore blocking and surface deposit phenomenon [76].

Humic acid (HA) or fulvic acid (FA) is a common compound in surface water and seawater, thus introducing a cake layer on the membrane surface [77]. The compound can lead to a severe fouling phenomenon. Besides organic matter, inorganic compounds such as mineral salt (CaCO_3_, CaSO_4_, BaSO_4_, SrSO_4_), metal ions may also introduce fouling and scaling. Generally, the severity of foulant is depended on the feed water of the membrane system. Usually, their proportion is 50% organics, 30% colloidal substance and 20% of mineral. Although minimal chemical component is applied in a membrane system, the concentrated retentate stream typically has higher concentrations of contaminants. Depending on the type of contaminants, the concentrate solution can either be disposed of through dilution, deep well injection, spray irrigation, or disposal in the municipal sewer.

### 2.6. Mitigation of Fouling in Membrane

Promising research efforts on membrane manufacturing focuses on finding suitable membrane materials with high chemical stability in an aggressive environment and solving the fouling problems. These can yield significant improvements in membrane performance, which are commonly constrained by the permeability–selectivity trade-off. Current research trends aiming to minimise fouling are moving in three directions: (a) modification of membrane using antifouling materials; (b) installation of pre-treatment system, which highly depends on feed content in which the pre-treatment system is mainly used to prolong the membrane’s life; (c) use of physical and chemical techniques in which backwashing and flushing procedures are used to recover the flux loss. In some cases, the loss is irreversible, and the only option is to replace the membrane with a new one. This leads to an escalation in the operating cost of the membrane system.

#### 2.6.1. Membrane Modification

**Figure 4:**
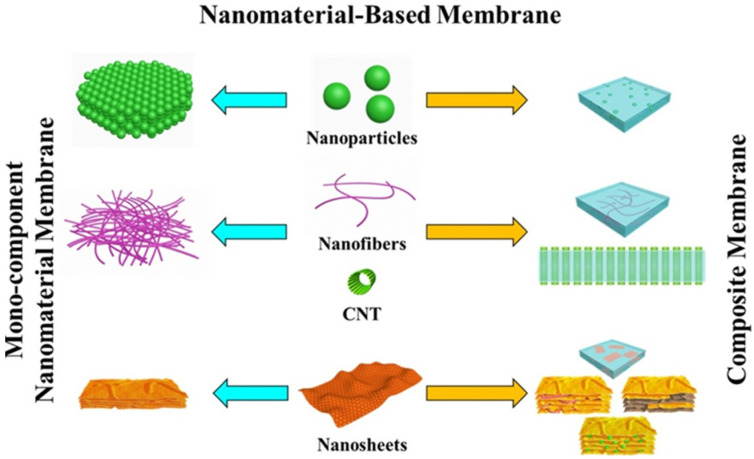
Nanomaterial-based membrane. Reprinted from [78] with permission from Elsevier, 2017.

Membrane properties are generally described as pore size, hydrophilicity, surface charge, chemical stability, thickness, mechanical strength, and thermal resistance. These properties vary based on membrane materials, structure, form, and application. With the recent progressions in nanomaterials and advanced membrane fabrication methods, simpler and reproducible surface modification techniques such as coatings, grafting and polymerization have been proposed. This membrane modification aims to increase fouling resistance, improve selectivity, and enhance the lifetime of the membrane. Nanomaterials are single materials sized between 1 and 100 nm and can be in the form of nanoparticles, nanofibers, two-dimensional layer materials, and other nanostructured nanomaterials (Figure 4). They exhibit excellent chemical and physical stabilities and have an enormous surface area, allowing extraordinary permeation properties. These nanomaterials might also provide additional antibacterial, antifouling and photocatalytic activity, which opens a new path to ultra-fast and highly selective membranes for water purification [78].

**Figure 5:**
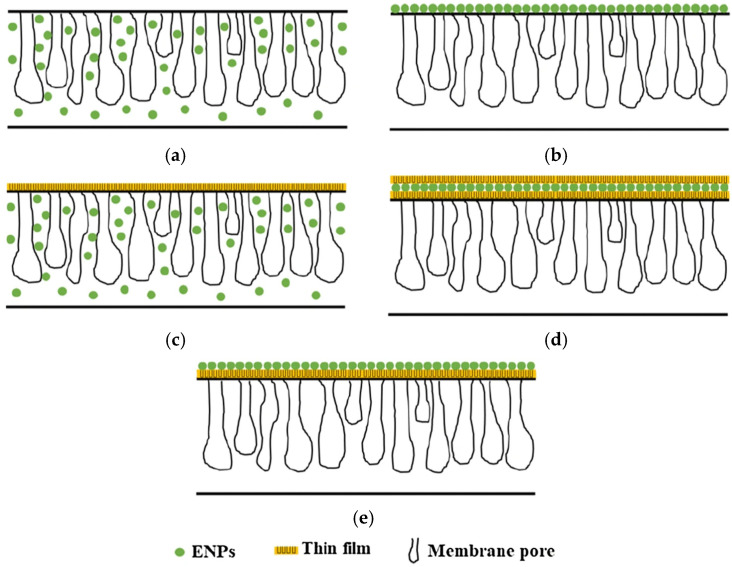
Design of nanocomposite membrane. (**a**) conventional nanocomposite, (**b**) surface coated membrane, (**c**) TFC with nanocomposite substrate, (**d**) thin film nanocomposite, (**e**) surface coated TFC. Reprinted from [81] with permission from Springer Nature, 2019.

A number of well-studied antimicrobial nanomaterials include titanium oxide [79], gold (Au), zinc oxide (ZnO), silver (Ag), carbon nanotube (CNT), graphene, and graphene oxide [80], have been utilized in various type of membrane-based water treatment system. The advances in nanotechnology and engineered nanomaterials presented a leapfrogging prospect to the next-generation membrane-based water treatment systems with a more affordable price and better purification efficiency. The nanomaterials can be integrated into the membrane to boost the efficiency and physicochemical characteristics by depositing them on the membrane surfaces or embedded into the matrices. Generally, there are five different nanocomposite membranes, including typical mix-matrix nanocomposite, thin-film nanocomposite, thin-film composite with nanocomposite substrate, and surface located nanocomposite [81] (Figure 5).

Despite significant progress on the development of nanocomposite membranes, several challenges need to be overcome for future scale-up manufacturing and application. Typically, the cost of most of the nanoparticles is high and requires a multi-step synthesis procedure, which results in poor reproducibility. Besides, it is challenging to ensure the homogenous distribution of nanoparticles in the polymeric matrix due to its tendency to highly agglomerate and incompatibility with the polymer matrix [82]. The agglomeration is caused by strong interactions between the nanoparticles and weak polymer–nanoparticles interfacial interaction, which cause the membranes to be vulnerable to defects and have poor separation efficiency. Therefore, it is vital to optimize nanofiller stability in the host polymer by establishing adhesive interface compatibility between the nanomaterials and polymer matrix [83]. Detailed investigation of the long-term stability of nanocomposite membranes is vital as poor adhesion of nanomaterial inside the polymer matrix or on the surface of the membranes could lead to the loss or leaching of nanomaterials during the filtration process. This will not only alter the membrane performance but can cause secondary contamination of the water [84]. Until today, the environmental and health side effects of nanomaterials for water treatment have not been systematically concluded [85].

#### 2.6.2. Pre-Treatment

**Figure 6:**
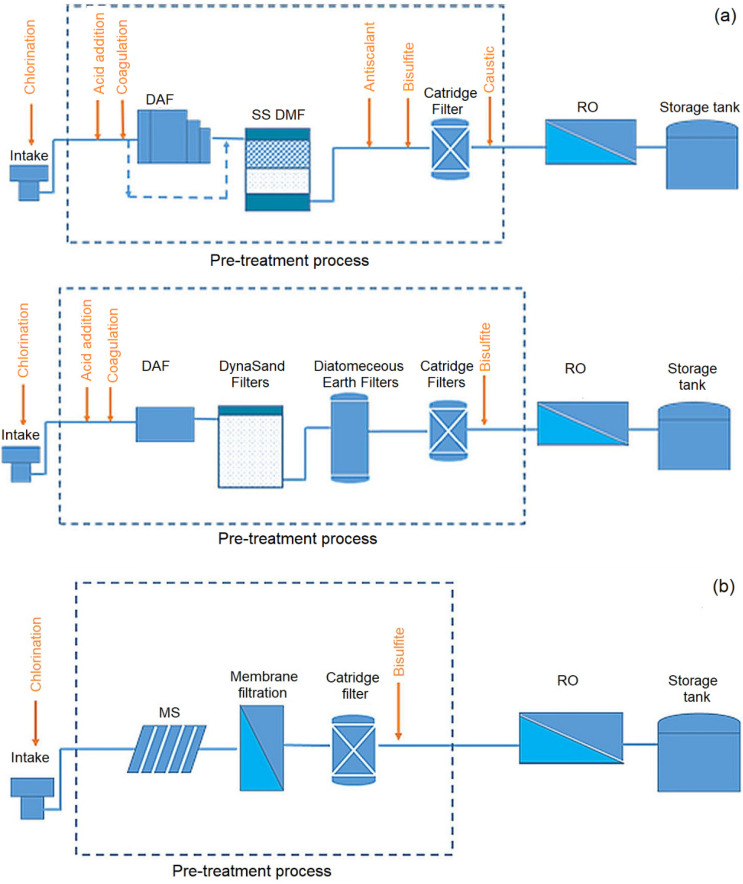
Pre-treatment processes: (**a**) Conventional pre-treatment process and (**b**) membrane-based pre-treatment process. Reprinted from [87] with permission from Elsevier, 2019.

The appropriate way to control irreversible fouling is through preliminary treatment of raw water prior to entering the membrane filtration system. A number of hybrid- and integrated-membrane systems such as coagulation, adsorption, biological filtration, oxidation and membrane bioreactors have been proposed [86]. The pre-treatment system’s choice is highly dependent on feed water characteristics, whereby every foulant has its unique method to selectively remove the foulant. Commonly, a pre-treatment system is beneficial for surface water filtration compared to underground water due to large amounts of contaminations. Several MF or UF systems have been utilized as a pre-treatment system to maintain the consistent and reliable operation of a seawater reverse osmosis plant (Figure 6) [87].

Pre-treatment by coagulation can selectively eliminate charged particles such as colloidal particles and metal ions. Alum is used as a coagulant to coagulate charged particles and settled down the foulant, while an activated carbon filter can be used to remove coagulated substances to produce feed that has the lowest colloids content [88]. A more advanced pre-treatment process using hydrogen peroxide (H_2_O_2_), ozone, UV radiation, and photocatalysts allows the molecular structure and properties alteration of the foulant due to the decomposition of organic pollutants. Consequently, low organic loading and biological fouling can be observed. Combined membrane systems are particularly useful for the treatment of surface water, which in contrast to underground water, is often described by the existence of large amounts of contaminations.

The application of the hybrid system combining adsorption on powdered activated carbon (PAC) with UF/MF to treat natural waters is more efficient than the process of unit membrane filtration [89]. The addition of carbon increases the efficiency of the membranes and the effectiveness of contaminations removal. The membrane acts as a physical barrier, inhibiting the passage of PAC, and thus the organic compounds which have been adsorbed on PAC are retained. This means the substances that lead to fouling are entirely retained by the PAC and do not deposit on the surface of the membrane.

Besides coagulation and adsorption on activated carbon, ozonation is also applied for the treatment of potable water. The purpose of ozone is to decrease the fouling phenomenon and quality of produced water as well as to increase the membrane lifetime [90]. The performance of an integrated coagulation–ozonation–ceramic UF-activated carbon filtration was evaluated for drinking water treatment from the micro-polluted surface water in southern China. It was discovered that the in situ ozonation in the membrane tank improves the removal efficiency for multiple contaminants, thus reducing the membrane fouling.

**Figure 7:**
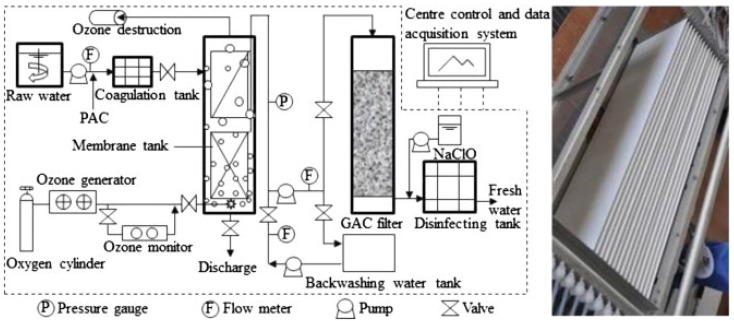
Schematic representation of the pilot-scale hybrid process for drinking water treatment. Reprinted from [91] with permission from Elsevier, 2014.

A higher degree of eliminating organic substances in potable water production can also be achieved by combining the filtration on a biologically active bed with membrane filtration. A 20 m^3^/day pilot-scale ozonation, ceramic membrane filtration (CMF) and biologically active carbon (BAC) filtration was utilized for indirect potable reuse, aiming for wastewater reclamation (Figure 7). The degradation rate of trace organic compounds was found to be more than 96%, and in situ ozonation was observed to be more efficient to degrade the organic pollutants and fouling due to higher residual ozone concentration in the tank [91]. Besides, the system has a better removal rate of ammonia and N-nitrosodimethylamine from the ozonated water. For denitrification of nitrate (NO_3_^−^)-polluted drinking water, membrane bioreactors to denitrify the water have been proposed as an alternative to biodegradation and filtration on sand beds or adsorption on activated carbon as it can provide complete retention of the biomass [92].

#### 2.6.3. Post-Treatment

**Figure 8:**
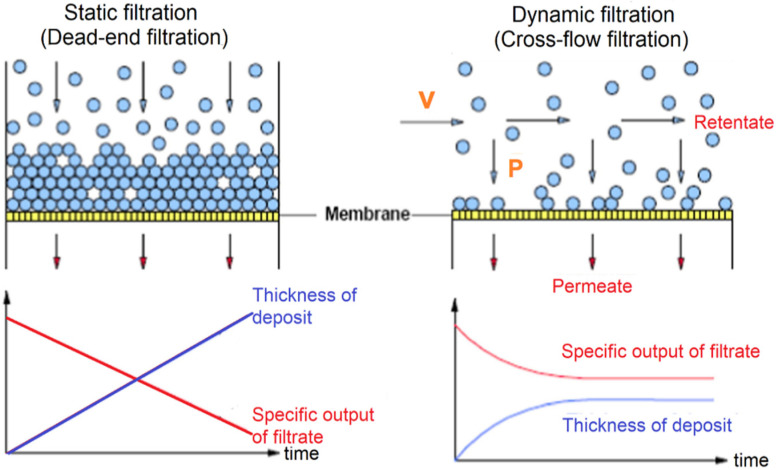
Membrane flow configurations and fouling formation [93].

Membrane systems can be operated in “dead-end” or “direct” mode, in which it has one feed stream and one filtrate stream [93]. In a dead-end manner, contaminants in the feed stream accumulate on the membrane surface and are held in place by hydraulic forces acting perpendicular to the membrane, producing a cake layer. The cake layer is typically removed from MF/UF systems through backwashing. The membrane will be replaced once the system faces a significant flow or transmembrane pressure (TMP) drop due to irreversible fouling. “Cross-flow” or “tangential flow” is operated by utilizing a high-pressure feedwater flow across the membrane. The solution is divided into two parts: (i) permeate, where a stream passes across the membrane, and (ii) retentate, where the remaining fluid flow on the membrane surface without separation or filtration. The retentate is usually concentrated with all rejected contaminants. The crossflow helps in maintaining a constant permeate flowrate and prolongs the membrane life by reducing irreversible membrane fouling (Figure 8).

A backwash process is typically carried out to eliminate contaminants collected on the membrane. It is conducted when there is an increase in TMP and/or a decline in permeate flux [94]. The direction and flow from the backwash will dislocate the contaminants from the membrane surface and washed out through the discharge line. A 5–10% reduction of system productivity can be expected after the backwash due to the volume of filtrate applied during the backwash. This indicates that although backwashing could enhance the fluxes, the complete removal of foulants could not be obtained alone through the backwashing process [95]. As a result, a chemical cleaning process needs to be utilised for inorganic scaling or foulants that cannot be removed by backwash [96]. Chemical cleaning is performed separately and in staggered manner for each membrane unit to minimize the number of units undergoing cleaning at one time.

The foulants that the backwash or chemical cleaning process can eliminate is identified as reversible fouling. After a particular time, the membrane system will face irreversible fouling, where the foulants cannot be eliminated through backwash and chemical cleaning [97]. Chemical cleaning agents are categorized into alkaline, acids, metal chelating agents, surfactants and enzyme [98]. Each chemical is targeted explicitly for a specific form of fouling. Strong caustic bases are typically utilized for dissolving organic material [99], while citric acid can be used for inorganic scaling [100]. Detergents and surfactants might be the best option for organic foulants that are difficult to dissolve [99]. Frequently, a combination of various chemicals might be utilized owing to the presence of numerous types of foulants in the water source. Table 1 summarizes the general chemical cleaning used for various type of fouling.

Polymeric-based membranes are known to be less tolerant to chemical cleaning than the inorganic ceramic membrane [109]. Thus, the chemical used for the cleaning and the regularity of the cleaning should be decided appropriately. For potable reuse application, the cleaning can be scheduled as frequently as once per day to once per month, depending on the quality of wastewater that need to be treated. In general, a clean-in-place (CIP) practice means the cleaning was carried out when the membrane modules remained within the membrane unit (in situ) [110]. A high velocity of cleaning solution is re-circulated through the membrane system at elevated temperature to create scouring action and to enhance the foulant’s solubility. A soak and flushing cycle was then conducted to eradicate residual traces of the cleaning solution. The processes may be undertaken several times using different cleaning solutions for multiple types of foulants. As compared to regular backwashing, chemical cleaning is performed only when necessary. As for MF and UF systems, the chemical cleaning will only be performed when the productivity rate cannot be restored using a backwash process. However, for NF and RO systems, chemical cleaning is performed when the flux decreases in the range of 10–15% or when the differential pressure increases more than 50%.

### 2.7. From Laboratory to Commercialization of Membrane Technology

One of the main criterium for consideration of new technology is its significant advantages over the conventional system. Membrane technology has increasingly emerged as a sustainable solution for water treatment, and considerable efforts have been made to enhance efficiency to attract investments. This could include lowering capital, operations and maintenance costs, simpler operation, better water quality produced and reduction in waste production. Until today, the main hurdles to large-scale implementation of membrane systems are its capital and operational costs. Constant innovations in the design of membrane systems aim to reduce the capital and operational cost to make it competitive compared to conventional treatment processes.

Scaling up the lab-scale membrane manufacturing and membrane operation for field performance testing remains challenging, particularly in ensuring the consistency in membrane quality for large volume processes, effective membrane module design and fabrication techniques. Besides, it is not easy to predict harsh operating conditions with possible field contaminants fluctuation during the field operation. As a result, all these challenges need to be addressed appropriately to ensure the membrane can be effectively applied as per the intended field specification. The challenges are divided into three sections: (1) Membrane manufacturing scale-up, (2) Module development and (3) Commercial-scale demonstration challenge. As for membrane manufacturing scale-up, many iterations and detailed characterization are required to evaluate the membrane’s formulation that yields the best performances. It is also vital that the manufacturing of membranes should be reproducible without significant changes to the membrane structure, and the formulations should be scalable for manufacturing lines. The repetition of formulation development and characterization will add more cost, besides the cost of raw materials. Issues related to the environment, particularly the selection of solvents and chemicals involved in membrane manufacturing, should be considered. Long-term mechanical and chemical stability in process environments should also be carried out. As for module development at the pilot scale, leaks and sealing issues should be avoided to ensure reproducible QA/QC. During the assembly of the module before the performance investigation, the issue related to membrane integrity should be considered. Verification at the demonstration-scale level, especially for different water sources, should be carried out to validate the potential of the membrane system. During testing of a commercial membrane system, pressure drop and mass transfer issues should be investigated thoroughly.

## 3. Feasible Membrane Technologies for Water Treatment in Developing Countries

Many developing countries in Asia and Africa face shortages of clean water supply to meet the demand [111]. Most of these countries are still using conventional water treatment systems extensively due to the low capital and operational cost. The consumers in these developing countries often pay much lower water tariffs than those living in developed countries, as the production cost of treated water with the conventional system is much lower. The affordability of the consumers becomes one of the biggest obstacles for the country to move forward with the more advance membrane-based filtration systems.

The increase of human activities such as industrialization and land development causes pollution levels to ascend drastically [112]. This poses a challenging situation for the conventional water treatment system to produce the desired treated water quality. Higher loading of contaminants and inconsistent raw water qualities will affect the treatment efficiency of the conventional system. Some of the operators of these systems would have no choice but to stop the water treatment plant operation if the desired treated water quality is not achieved due to the fluctuating quality of the raw water. Under these circumstances, the membrane filtration system becomes an alternative option to consistently produce excellent treated water quality.

### 3.1. Membrane System for Clean Drinking Water Production

**Figure 9:**
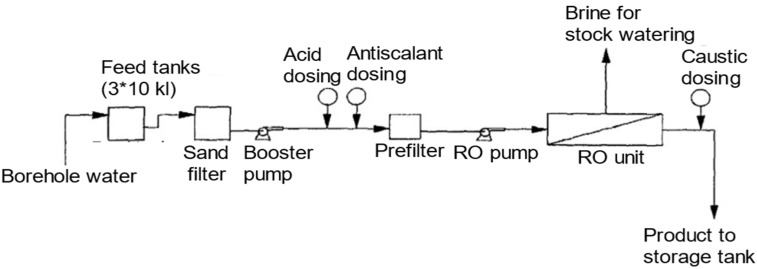
Flow diagram of the RO plant in South Africa. Reprinted from [52] with permission from Elsevier, 2003.

Some of the most commonly used large-scale membrane water treatment systems are ultrafiltration (UF) and reverse osmosis (RO) [113]. Seawater reverse osmosis (SWRO) plants commonly utilized UF as pre-treatment and RO for desalination [114]. As for surface freshwater, typically, only UF is used for the solid–liquid separation process. Compared to the RO process, treating freshwater is much more economical than sea water due to the lower pressure required for the UF [115]. Most developing countries are more inclined to use freshwater as their raw water source in large-scale water treatment plants for economic reasons. Rural villagers in low-income countries such as Kenya have resorted to various rain harvesting methods to fulfil their daily freshwater needs [116]. In the rural area of South Africa, borehole water was found to have high nitrate–nitrogen and salinity, which is not safe for human consumption. Therefore, the potential of the RO system to produce clean water was investigated (Figure 9). It was found that the RO process is effective for water denitrification and water desalination. The capital cost for 50 m^3^/d output RO plant was found to be around the USD 29,900, while the operational cost was approximately USD 0.50/m^3^ [52]. This cost was considered high, and thus chemical dosing or blending borehole water with RO product water are needed.

**Figure 10:**
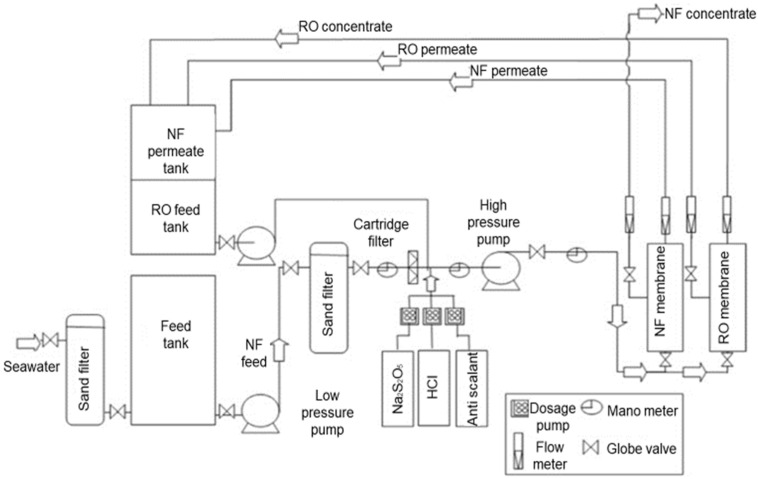
Flow diagram of the NF + SWRO integrated desalination process. Reprinted from [117] with permission from Elsevier, 2015.

Kaya et al. [117] proposed the use of NF as a pre-treatment stage of reverse osmosis (RO) process for seawater desalination system located at Urla Bay, Izmir (Figure 10). It was found that the scaling issue on SWRO membrane decreased significantly, which led to the reduction of desalination cost. Although permeate flux and recovery enhanced, the rate was greatly affected by the type of NF membranes due to the difference in pore size.

Although large-scale membrane water treatment systems are still at the infant stage in many developing countries, some have achieved quite impressive results [68,116,117]. These membrane filtration systems usually complement or support the existing conventional system to increase the treated water capacity to meet growing demand. Large-scale membrane systems such as RO is impractical for economically challenged developing countries, particularly for populations living in rural and remote villages [118]. Such systems would require substantial advanced infrastructures and electricity supply for operations. A small-scale solar-powered membrane water treatment system is much more feasible for these rural villages [119]. It is still an uphill task for many developing countries to develop the capital and operational budget to maintain these facilities for the rural areas.

There is a vast potential for membrane-based desalination systems such as RO to replace the energy-intensive thermal/distillation technology [120]. This is mainly due to the lower energy consumption and higher efficiency of the RO systems. Recent technological developments in polymeric and ceramic type membranes have further propelled the potential for large-scale desalination systems with much lower operational costs [121]. Growing global population and rapid industrialization have pushed many countries to desalination and wastewater reclamation technology to fulfil the water demand. Some of the critical issues of RO membrane fouling have been addressed with membrane surface modifications to mitigate the problem [122]. One of the significant challenges of the desalination system is to ensure the affordability of the treated water to the consumers or end-users. More than 50% of the installed desalination RO plants are currently located in the United States of America and the Middle East, which is home to less than 5% of the global population [123].

Natural surface water remains one of the most widely used raw water sources for large-scale water treatment plants [124]. Many UF membrane systems have been utilized to process raw surface water into safe drinking water for public use [125]. Rapid industrialization in China for the past two decades has propelled the emergence of numerous research and development (R&D) on large-scale UF membrane water treatment plants [23,70]. The applications of UF systems are particularly suitable for highly urbanized cities that are facing the scarcity of land for the treatment infrastructures. It has been reported that UF systems take up almost 70% less footprint compares to the conventional sand/media filtration system [5]. With the distinct advantages of being more compact and having higher filtrate quality, UF membrane systems have become another feasible alternative worth considering in many metropolitan cities.

Table 2 lists out several membrane systems that have been used in developing countries. To date, small-scale membrane systems are typically used to produce potable water from brackish or seawater. Most studies indicate that a pre-treatment is critical to ensure that the feed water is compatible with the membrane system. Not only that, the use of a pre-treatment system before the membrane system enhances the efficiency and life expectancy of the membrane by decreasing the fouling and scaling issue. Some of these membrane-based systems treat contaminated water, which further substantiate the improved water quality obtained using this technology. As cost is a huge concern in most developing countries, surface water or freshwater will still be the priority, as it costs much less for treatment compared to seawater.

### 3.2. Cost Analysis of Membrane Systems

It has been widely accepted that the membrane system incurred much higher capital and operational expenditure compared to the conventional system [63]. The most significant saving of the membrane system is on the smaller footprint required [141]. In high-dense population urban cities, land acquisition to build a water treatment plant is substantial and incurs a significant cost. Developed countries have taken full advantage of the small footprint to build compact large-scale membrane water treatment systems to fulfil the demand for highly populated urban cities [142]. Unfortunately, the smaller footprint might be the only significant cost saving for these systems compared to the conventional water treatment systems. It is widely documented that capital and operational expenses for large-scale traditional water treatment systems is much lower and thus often become the most preferred system in developing countries [143]. One of the most significant operational expenses for large-scale water treatment systems is the electricity consumption. Industrial-scale UF membrane water treatment systems could cost more than 20 times in electricity consumption compared to the conventional system using the same source of raw water as feed. It has been anticipated that due to the mass production and competition among membrane manufacturer, more affordable and higher quality of membrane will be made available in the near future. In developing countries, small to medium scale membrane-based water treatment systems are confined to privately owned factories to cater to production needs. This is primarily due to the insufficient clean water supply from government-owned facilities for these factories.

Over the last few years, the cost of manufacturing polymeric membranes has reduced substantially due to better production techniques and economies of scale. It has been reported that an industrial-scale UF membrane plant capital cost is only about 6% more than the conventional system. However, the estimated electricity cost for the UF system is more than 20 times higher [5]. One of the hidden costs of the membrane system is the periodic maintenance required. Due to the more complicated automation, highly trained technicians and engineers are often stationed at these water treatment plants, which incurred significant maintenance costs. In addition, many mechanical equipment (e.g., pumps, valve actuators, electrical relays, etc.) are installed to enable the complete automation for periodical cleaning of the membrane. Unlike the membrane system, which requires backwash or cleaning every few hours of filtration, the conventional system could operate for days before a backwash is initiated [144]. This allows less costly automation installation, and the conventional system could be operated under manual mode to reduce the overall capital and operational expenditures.

A detailed analysis between industrial-scale UF and conventional water treatment systems for raw surface water has indicated that the overall cost of the UF system is still much higher [5]. Table 3 summarizes the various cost incurred in general between the two water treatment systems.

Table 3 implies that as land scarcity becomes more apparent, especially in high-density urban cities, the UF membrane system would become a more attractive solution. A land-scarce developed nation such as Singapore has adopted many large-scale membranes water treatment plants to fulfil their water needs [21]. It is estimated that more and more developed countries shall face similar land scarcity issues due to the mass migration to cities or urbanization.

### 3.3. Affordability, Supply and Demand for Clean Water

Most developing countries indicate a much lower per capita income compared to developed countries [145]. This shows the less spending power on basic necessities such as water and electricity utilities. The governments in these countries have little choice but to continue large-scale conventional water treatment systems to ensure the affordability of the consumers. The overall operational cost of these water facilities are mainly derived from revenue collected from the consumers based on the stipulated water tariff imposed [146]. Affordable water tariff is generally defined as less than 5% of household income spent on water bills [147]. In Southeast Asia (SEA), in developing countries such as Malaysia and Indonesia, water tariffs are much lower compared to developed nations such as Singapore, although these countries are close neighbours. Large-scale membrane systems are used extensively in Singapore for clean water production, requiring much higher costs than conventional systems.

It is estimated that the water demand of most developing countries shall keep on increasing as they move forward with more industrialization activities and population growth [148]. This has posed a strain on the existing raw water resources and water treatment plants to produce sufficient treated water for the country needs. When the relatively unpolluted raw water sources are scarce, the next step is to look for raw water with much higher contaminant loading but which is still abundantly available. Due to the limitations of the depth filtration mechanism in the conventional media sand filters, it is incapable of handling some contaminant removal to ensure an effective solid–liquid separation process [149]. A membrane system such as UF offers a feasible alternative to handle high suspended loading and yet provides a good quality filtrate through the surface filtration mechanism [150]. This enables raw water with much higher contaminant loading to be processed with less retention time in the system.

The correlation between demand, supply and affordability has posed a serious issue to many developing countries. The demand can be met by constructing more feasible water treatment systems to produce the supply. Nevertheless, due to limited “unpolluted” raw water sources, a lower grade of raw water sources has to be used. That shall increase the overall production cost and inevitably increase the water tariff. Raising water tariffs significantly will have a chain of economic repercussions, especially for the developing countries.

## 4. Challenges and Opportunities of Membrane Technology Implementation in Developing Countries

Improving the standard of living for the population in developing countries remains an uphill task without sufficient clean potable water supply for all, especially in rural areas. This research paper intends to highlight the feasibility of various membrane technologies for water treatment in these countries. There are many limitations in developing countries to seek more reliable alternative water treatment processes such as membrane systems. One of the main challenges for large-scale membrane systems in these countries is the overall cost incurred against the affordability of consumers. Many stakeholders (e.g., government and private sectors) are keen on adopting large-scale membrane water treatment systems in developing countries, looking at the positive economic, rapid industrialization, and population growth. Based on the current consumer’s affordability in most developing countries, the water tariff needs to remain low to accommodate the population income. Adopting a large-scale membrane water treatment system will undoubtedly increase the water tariff significantly. A balance needs to be reached to ensure that clean water supply is sufficient and water tariff remains affordable to most people in these countries. One option is to allow the treated water from these large-scale membrane water treatment plants to be supplied to only the industry or factory for their manufacturing process [151]. Higher water tariffs could be imposed on these profit-making industries to encourage these factories to install their own water treatment facilities in order to fulfil their manufacturing demand. This would free up more resources from the municipal water treatment plants to cater for domestic users. Government intervention is required as a proper guideline has to be drawn up to ensure compliance [152].

Decentralized small-scale water treatment systems have been a feasible solution for many rural villages with a small population in developing countries. Small-scale UF systems have been utilized as a direct filtration process without using any coagulant or chemicals for Malaysia’s rural river water source [119]. In another project, a membrane-based water treatment system was set up and monitored in Tanzania’s rural community for over 9 months [153]. This low-cost system has shown promising results as a decentralized water treatment pilot plant for other similar rural villages. The major challenge of these decentralized systems is the long term operational and maintenance cost. Local governments have to formulate a workable solution to ensure the sustainability of rural water supply schemes.

Direct filtration using a low-pressure membrane such as UF is suitable for raw water with low turbidity and suspended solids without any coagulant required [68]. Unfortunately, most raw water source fluctuates in quality and a more robust pre-treatment need to be in place to prevent severe membrane fouling. A hybrid membrane process has been vigorously studied to ensure higher contaminant removal and mitigate membrane fouling issues [154]. In this process, activated carbon, which is commonly used as filtration media in the conventional water treatment system, is added prior to the membrane filtration process. This process has the advantage of maintaining a relatively small footprint and yet provides an extra precaution to reduce membrane fouling even with relatively polluted raw water. An additional operational cost will be incurred for the continuous addition of the activated carbon, which requires a feasibility study for the long run.

One of the highest operational costs for membrane water treatment systems is electricity utilization [155]. Renewable solar power has been considered a viable energy source to reduce the carbon release in producing electricity [156]. The notion of having “free” electricity for the pressure-driven membrane filtration process would be of interest to many large-scale facilities stakeholders. In most cases, a significant amount of initial capital expenditure will have to be disbursed for all the necessary solar panels and ancillary equipment before converting solar energy to electricity could be achieved [157]. Instead of paying electricity as an operational cost, solar energy harnessing facilities have become a capital cost. The feasibility of using renewable energy in large-scale membrane water treatment systems will be determined by various factors and differs for each developing country.

It is an uphill task in most developing countries to provide basic utilities such as electricity and clean water, especially to the vast rural populations [158]. Infrastructure developments in these countries are concentrated in urban cities whereby all the major economic activities thrive. Both government and private sectors rely on each other to ensure a conducive business environment in the towns for mutual benefits. As for the rural villages, infrastructure developments are hampered due to the lack of business activities. Governments in developing countries would need to build up their financial coffers to upgrade the basic utility supply for rural villagers. Most developing countries have plans to provide these utilities, but financial constraints are still prevalent in many regions.

### 4.1. Current Scenario of Water Treatment Facilities in Developing Countries

Many developing countries are still struggling to provide clean water to some rural areas lacking water and electricity supply [158]. Due to the low population density and per capita income, supplying these utilities in rural areas is challenging. Many rural villagers can only obtain raw untreated water from ponds or rivers for daily usage because of the lack of piped water supply in these remote areas [159]. It is a challenging task to ensure that all areas in these developing countries have access to clean piped water with limited resources from the government.

A conventional water treatment system is still the most cost-effective process, provided a relatively “unpolluted” raw water source is available [160]. Due to the constant increase of water demand caused by thriving industrialization in developing countries, these raw water sources are slowly depleting. Many cases of raw water contamination have caused these large-scale conventional water treatment plants to be shut down as these systems are not designed to removed other harmful dissolved pollutants [161]. Membrane filtration systems offer more robust removal efficiency, especially with the combination of UF and RO membranes [162]. Developed countries such as Singapore have even used membrane systems to recycle sewage water into high grade processed water for various industry applications [60]. The dwindling “unpolluted” raw water sources in developing countries are slowly pushing them to adopt a more robust system to ensure the sustainability of their clean water supply. Due to the relatively high cost incurred in developing countries, small and medium-sized membrane plants are mostly installed at manufacturing factories. Most of these factories rely heavily on clean water supply for their manufacturing process but piped water supply from local authorities might not guarantee both quality and quantity to fulfil their demand. Thus, it is quite common for these factories to source raw water (e.g., groundwater or nearby stream) and carry out the water treatment process themselves at their premises with the authority’s approval.

Consumption power of the people in developing countries is usually much lower due to their below-average per capita income. The basic utilities such as water and electricity tariffs are usually kept low through government intervention (such as subsidy) to ensure the people’s affordability [163]. In general, the conventional water treatment system is the most affordable process to provide clean water according to World Health Organization (WHO) standards. Many of these developing countries are still allocating a vast sum to build large-scale conventional water treatment plants to fulfil the country water demand. The water tariffs in these countries are much lower than in developed countries in the same region. The sustainability of these water treatment plants depends on the revenue collection based on the approved water tariffs.

### 4.2. Technology Transfer from Developed/Advanced Countries

Developing countries face two main challenges in adopting large-scale membrane water treatment systems. The first is the relatively low water tariff as well as the affordability of the people. It is commonly accepted that capital and operational expenditure for a membrane system is much higher than the conventional system. Thus, it will incur a higher water tariff to ensure economic sustainability [164]. The second obstacle is the lack of infrastructure and experts to support the operation of these advanced systems. Membrane systems require highly automated operations with competent personnel. Most of these membranes and supporting equipment are unavailable in developing countries and are imported from more advanced or developed countries overseas. The willingness and co-operation between these countries (developed and developing countries) is paramount to ensure a successful technology transfer of these advanced water treatment systems.

One of the possible solutions is to promote the privatization of water supply to prospective investors. The build, operate and transfer (BOT) business model allows minimal financial burden from local government to develop water infrastructure for the public [165]. The conventional and membrane-based water treatment systems both have their distinctive advantages and weaknesses. A detailed feasibility study of BOT model for developing countries is always necessary because it involves long-term investment. Investors have to ensure the water tariffs have to be affordable for the public and yet the revenue collected will enable them to make a continuous profit. Striking such balance is another uphill task for all the stakeholders involved. Both technology transfer and investment from foreign investors might be a possible solution to ensure the sustainability of large-scale membrane water treatment systems in developing countries.

## 5. Current Trends and Future Outlook

### 5.1. Improvements in Membrane Modules and Membrane System Configurations

Researchers are looking into other strategies to overcome fouling challenges and enhance membrane performances, including improved membrane module design and novel hydrodynamics. Typically, the design of membrane modules is plate and frame, hollow-fibre, tubular and spiral wound [166]. A hollow fibre module typically comprises a number of hollow fibres bundled together into an element forming a pressure vessel. The HF module presents high packing density and consistent permeate flow with minimal pressure drop, allowing 10 times more flux than spiral wound modules. In a spiral wound module, two membranes are separated by a feed channel spacer and placed where their active side facing each other is centrally connected to a perforated permeate collection coil. The feed channel spacer promotes turbulence conditions and permits the homogenous flow of feed water across the membrane [167]. Generally, two standard techniques to enhance module performances include design effective module with optimized flow geometry, known as passive enhancement and utilization of external energy such as bubbling, vibrations and ultrasound to prompt a high shear regime to minimize the fouling and concentration polarization phenomena, which is known as active enhancement [168].

**Figure 11:**
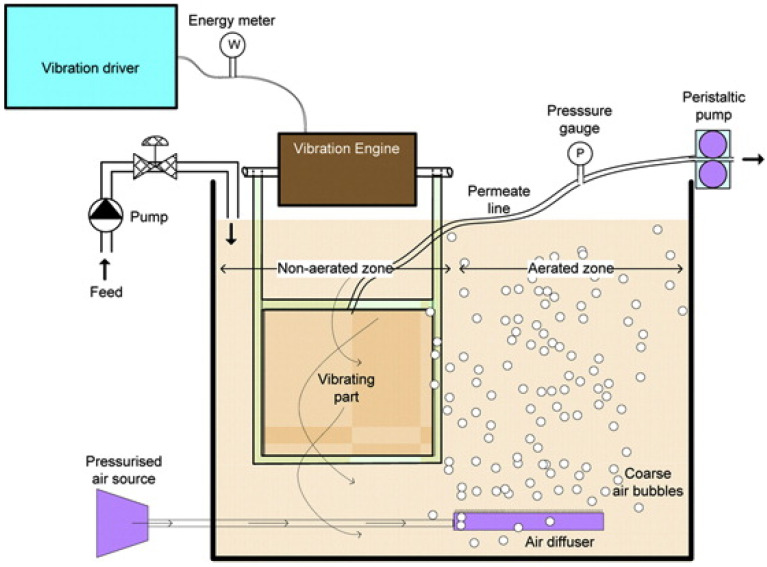
Schematic diagram of novel magnetic vibrating module (MVM) that offers high flux and lower degree of fouling. Reprinted from [171] with permission from Elsevier, 2012.

In a recent development, a rotating disc module was fitted with a UF membrane to control the thickness of the cake layer and ease membrane fouling through flow velocity improvement and shearing force in the presence of flocs [169]. The system revealed that floc-based cake layers could be efficiently controlled with module rotation, making them suitable for drinking water treatment systems. Another industrial dynamic filtration unit consists of disks or rotors rotating near the fixed membranes or rotating organic/ceramic disk membranes and vibrating systems [170]. A high shear rate can be produced with no significant feed flow rates and pressure drops, a feasible substitute for crossflow filtration. A novel magnetically induced membrane vibration (MMV) system was examined in a lab-scale MBR to replace the conventional submerged membrane module (Figure 11). Instead of using coarse bubbles aeration for the sheer production, an intermittent vibration technique was utilized, leading to energy saving [171].

**Figure 12:**
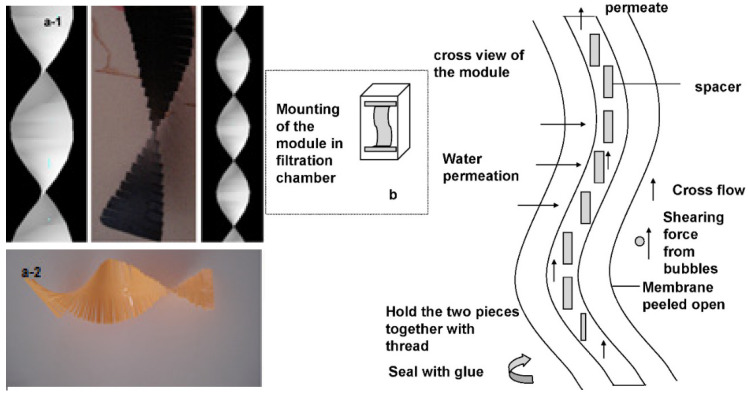
(**a-1**,**a-2**) the fish-bone or broom-like structure of spacer (**b**) cross view of helical membrane and its mounting in filtration chamber. Adapted from [172] with permission from Elsevier, 2010.

A helical membrane module unit comprises an inside helical spacer and an outside cover to reduce filtration resistance, reduce fouling, and enhance permeate flux [172]. The helical membrane consists of two sheets of terylene filter cloth, with a pore size of around 22 µm and a total effective area of 0.022 m^2^. The membrane was supported on a plastic spacer resembling DNA helix that acts as a “stirrer” and “rotating ladder” (Figure 12) for mass transfer improvement through vortex mixing and turbulence enhancement. The new module was found to decrease filtration resistance because of vortex mixing and intensified turbulence at the membrane surface. As a result, the expense per amount of mass transferred can be minimised, which can be directly translated into energy consumption and module manufacturing cost.

**Figure 13:**
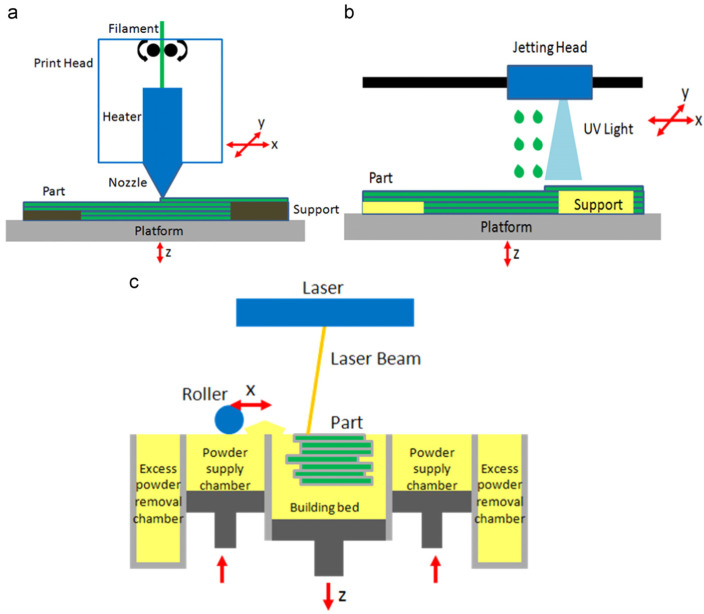
(**a**) Solid-based, (**b**) liquid-based and (**c**) powder-based 3D printing technologies. Reprinted from [174] with permission from Elsevier, 2016.

Recently, surface patterning using a 3D printer was utilized to modify the membrane surface topography as a way to mitigate membrane fouling [173]. Besides the hydrodynamic effect, surface patterning can inhibit the deposition of foulants on the valleys when the particle size is bigger than the valley size or by modifying the particle crystallization entropy when the size is comparable. In addition, novel and innovative membrane module components can be developed quickly and at a low cost using 3D printing technology. The fabrication and optimization of membrane module elements could be promptly prototyped, which is unreachable using traditional manufacturing methods (Figure 13). The patterned surface permits the generation of eddies, and with the cross-flow velocity combination, the back-diffusion of foulant to the bulk liquid can be facilitated [174].

### 5.2. Development of Renewable Energy-Driven Membrane System

As for the development of system infrastructure for clean water production, energy use and the carbon footprint of water consumption have emerged as critical issues. As a result, the correlation between water and energy consumption, known as water-energy nexus implications, is integral to developing a sustainable and low-cost water purification system. The growth of water resources should not come with high-cost energy consumption. Due to the high energy and separation efficiency, membrane-based technologies have earned pervasive implementation in various water treatment processes. Ghaffour et al. [175] reviewed the challenges and potential applications of using renewable energy-driven desalination technologies. They indicated that solar-driven RO plants allow for the elimination of the fossil fuel dependency for producing adequate freshwater. However, the efficiency of the solar energy-driven system is highly linked with the design and arrangement of PV arrays, tilt angle and cleaning methods. An efficient system can ensure an uninterrupted system, increasing the distillate production and cut down the overall water production cost [176]. Elmaadawy et al. [177] investigated several types of the renewable energy system for large-scale RO desalination plants (1500 m^3^/d) and compared two off-grid scenarios with various combinations of hybrid power systems and diesel systems. The results demonstrate that the recommended system significantly reduces from 60–81.5% compared to existing diesel with respect to the net present cost, renewable fraction, cost of energy, and carbon dioxide emission, respectively.

Chew and Ng [119] investigated the feasibility of a solar-powered UF system at a rural village in Perak, Malaysia and the performances was compared with a sand/media filtration system. A solar-powered UF system can obtain a higher quality of treated water with less than 0.4 NTU turbidity and lower operating cost and carbon release. The use of cross-flow filtration operation mode eliminates a daily intermittent backwash sequence, which further simplifies the daily operational routine suitable for rural areas. However, one of the main challenges of a solar-driven system is related to energy storage capacities. The prospective for lithium-ion (Li-ion) batteries and supercapacitors (SCs) on a photo voltaic-powered RO membrane (PV-membrane) system was evaluated, and it was observed that average specific energy consumption (SEC) of 4 kWh/m^3^ with fully charged batteries is used to produce clean water [178].

### 5.3. Development of Alternative Pressure-Driven Membrane System for Desalination

In addition to using renewable energy with a traditional membrane filtration system, advanced membrane operations can be attractive for the production of renewable energy in the future and might shift the conventional perception of renewable energy sources. Seawater desalination is seen as one of the solutions to clean water scarcity. It allows a climate-independent source of drinking water and is increasingly being used to provide drinking water around the globe. More desalination plants worldwide are using RO technology due to its simplicity and low energy cost compared to flash distillation thermal processes. Besides RO, forward osmosis (FO) and membrane distillation (MD) are among the emerging membrane-based system appealing for desalination [179]. FO and MD have received significant attention from researchers in the membrane field and industry, as shown by the increased publications. Among the essential features of this third-generation desalination process is the ability of FO and MD to minimize the energy required, and the operating cost is much lower than RO [180].

**Figure 14:**
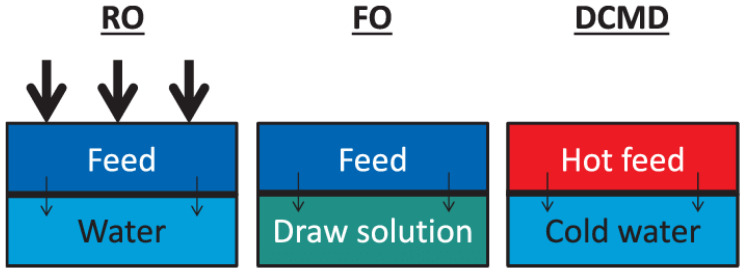
Working principles of reverse osmosis, forward osmosis and membrane distillation Reprinted from [185] with permission from Elsevier, 2018.

During the RO process, a high operating pressure (up to 1000 psi) drives the saltwater through a membrane. As a result, the energy use in RO is typically higher. Meanwhile, FO operates at low pressure, thus requiring lesser energy and low fouling compared to the RO process. In FO, the water in the feed solution (low osmotic pressure) freely flows through a selectively semipermeable membrane to the draw solution (high osmotic pressure) under the osmotic pressure difference. FO has been utilized in desalination and complex industrial streams [181]. When FO was used for high salinity brines (TDS > 70,000 mg/L), the recovery of feed water was found to be more than 60%, and the quality of treated water could meet the discharge quality criteria of surface water [182]. RO and FO typically use similar types and pore size membranes. In comparison [183], MD is a thermally driven membrane system using a microporous, hydrophobic, vapour-filled membrane. The MD is not entirely viable at a commercial level because of several reasons: (1) vague water production cost (WPC) due to high specific energy consumption (SEC), (2) lack of suitable membranes and modules, (3) membrane pore wetting phenomenon due to the use of unsuitable membranes, and (4) undefined long-term operation because of membrane fouling and/or scaling [184]. Figure 14 compares the working principles of RO, FO and MD.

### 5.4. Zero Discharge Liquid (ZLD)

Besides the water and energy shortage, lawful obligations for the discharge of waste and wastewater have forced the industry to change its liquid waste management approach to be more sustainable through integrated concepts. As a result, verifying the prospective applicability of zero liquid discharge (ZLD) for water treatment is essential. ZLD is a water treatment approach where all wastewater is purified and recycled, resulting in zero discharge at the end of the treatment cycle. ZLD relates residual output in terms of waste, wastewater and energy loss to the process input based on materials and energy, allowing the prospect of using a process cycle where wastewater treatment is envisioned for water recycling, considering the mass balance of materials other than water and the energy balance.

The environmental impacts of brine dumping and greenhouse gas (GHG) emissions from seawater desalination plants and wastewater treatment plants have been a rising concern due to water scarcity. An innovative and energy-autonomous (through solar energy) pilot SOL-BRINE system with a capacity to treat 2 m^3^/day of brine has been installed in Greece to achieve ZLD in a desalination plant. It was found that the recovery of water (> 90%) and dry salt (full recovery) can be achieved [186]. Recently, China and India have been among the countries that have implemented strict regulations that make ZLD as a necessary strategy for sustainable wastewater management and to protect freshwater resources. In China, the ZLD approach has been widely adopted in developing new power and chemical plants due to public protest. The government of India has established a draft policy where the textile plants need to install ZLD facilities if they are producing more than 25 m^3^ of wastewater effluent/day [187].

Although ZLD could minimize contamination and enhance clean water supply, one of the significant concerns of ZLD at the industry level is the high capital cost and intensive energy consumption [188]. As a result, membrane-based technology is theoretically an attractive approach that can be utilized to reach this aim. Several recent works have emphasized the use of a series of membrane processes as a possible way to accomplish ZLD at the industry. In Tamil Nadu, the Perundurai Common Effluent Treatment Plant is among the first plant in India that implemented ZLD to treat and manage effluents from several textile processing industries using RO and evaporator [189]. Although the cost of ZLD is slightly higher, the expenditure of the system is expected to be reduced through salt recovery.

One of the examples is the FO-based ZLD system in Changxing coal-fired power plant that is capable of producing high-quality permeate water for reuse in any industrial process and concentrating brines up to a high TDS concentration for further studies processing in a crystallizer [190]. However, not many works have discussed in detail the challenges encountered with ZLD approaches.

## 6. Outlook on the Adaptation of Membrane Filtration Technology by Developing Countries

Rapid industrialization in many developing countries is swiftly transforming these countries’ economic landscape. More factories will require a higher volume of clean water for product manufacturing to increase productivity. The increase in industrial activities would bring forth more income to the government in terms of tax revenue to improve public utilities such as clean water supply to the public and industry. Stakeholders or government officials would need to devise future plans to ensure the sustainability of their country water resources in the coming years. The depletion of good quality raw water sources from reservoirs such as rivers and lakes are prevalent due to pollution caused by human activities, particularly in rapidly industrialized developing countries. The conventional water treatment system utilizing sand or media filtration which most developing countries heavily rely on might not be able to handle the more challenging task of treating polluted raw water sources.

Membrane technology will be one of the most feasible alternatives to carry out the more challenging task of water purification due to its many advantages over the conventional system. Membrane filtration has a compact design and high degree of flexibility, and therefore the use of membrane systems is expected to rise in future. It has been a game changer utilized to convert seawater into potable water in desalination plants around the world. Nevertheless, the cost factor remains one of the biggest obstacles for its adaptation in developing countries. With the increased income of rapid industrialized developing countries and the more affordable cost due to technological advancement in membrane technology, it is envisaged that membrane systems will become highly feasible in the near future. The successful implementation of many large-scale membrane water treatment plants in advanced countries further substantiates the maturity of membrane technology for water purification.

From the global industrial complex perspective, the most significant issue is how to make membrane technology affordable in low-income countries with restricted access to RO technology. Perhaps, membrane systems’ capital investment and operating costs must be reduced significantly to make this viable. The utilization of solar energy might be able to mitigate some of the high operational cost for membrane-based desalination, but the high capital investment for the solar PV system remains a challenge for most developing countries. Freshwater sources will still remain the main sources of raw water for treatment in these countries, and desalination would be an alternative when freshwater resources have been depleted. This review intends to provide stakeholders and researchers with comprehensive information to evaluate membrane technology for water treatment, particularly in developing countries.

## Figures and Tables

**Table 1:** Chemical cleaning for various types of fouling.

Fouling Type	Chemical Cleaning and Findings	Ref
Municipal wastewater: Organic, inorganic and biofouling and microbial	NaOH-EDTA-SDS alkaline treatment and citric acid (pH 2) treatment. 70% of membrane foulants were removed by cleaning. Bacteria with excessive extracellular polymeric substance (EPS) such as Pseudomonas and Zoogloea were more resistant to chemical cleaning	[101]
Surface water: Organic, inorganic and biofouling	2% HCl and caustic 2% NaClO. Alkaline cleaning removed most of the microorganisms and organic foulants on both membrane’s external and inner surfaces. Acidic cleaning effectively removed the inorganic scales.	[102]
*P. granatum* (pomegranate) juice	1%w/w P3 Ultrasil 53 solution (a neutral enzymatic powder containing organic and inorganic surfactants). 90–95% of the initial water permeability was recovered	[103]
Humic acid and Sodium alginate mixture: Organic fouling	sodium hypochlorite (NaClO). Concentration as low as 1 mg/L and backwash time 30 s leads to flux recovery of 92.1%.	[104]
Sugarcane juice: biofouling	Acidic, alkaline, protease (i.e., trypsin), dextranase and lysozyme solutions. The use of enzymatic dextranase cleaning to degrade dextran foulant layer prior alkaline cleaning leads better removal rate.	[105]
Surface water and ground water with NOM: Organic fouling	0.1 M Citric acid, 0.1 M caustic NaOH, and 0.001 M surfactant SDS. Surfactant was not effective to remove high NOM content. High cross-flow velocity and longer cleaning time influenced the efficiency of caustic cleaning.	[106]
Domestic wastewater: Organic and biofouling	NaOCl and citric acid as the order. The organic foulants such as FA and HA and microbes (proteobacteria, Firmucutes, Epsilon bacteria and Bacteroides) were effectively removed by NaOCl	[107]
Boiler water: Inorganic fouling	HCl, H_2_SO_4_, H_3_PO_4_, nitric acid, citric acid, NaOH, potassium, EDTA, SDS and commercial dish washing detergents	[108]

**Table 2:** Membrane system used in developing countries.

Country	Water Source	Membrane System	Pre-Treatment (Capacity)	Conclusions	Ref
Malaysia	Surface water and Groundwater	UF	Nil (15,536 MLD)	Effective at removing heavy metals (Cr, Cd, Zn, Cu, Ni, and Pb from 92% to 100%) but expensive.	[126]
Turkey	Seawater	RO and NF-Desalination	NF (Not available)	NF could be an ideal pre-treatment step for the SWRO desalination to improve permeate flux and recovery by eliminating the scaling problem and reducing the cost of the desalination process	[117]
South Africa	Groundwater	Gravity driven UF	-Nil (5000 L/d)	The microbiological quality of the permeate was acceptable, and the integrity of the filtration membrane was still maintained after ten months. Total coliform removal (2419.2 to 7 cfu/100 mL) and *E. coli* and Enterococc: Complete removal	[127]
South Africa	Borehole Water	RO Denitrification and Desalination	3 dual media sand filters using 2.5-μm cartridge filter (50 m^3^/d)	RO effectively for water denitrification in a rural setting. Nitrate–nitrogen (reduced from 42.5 to 0.9 mg/L) and TDS of RO (reduced from 1292 to 24 mg/L)	[52]
India	Pesticide contaminated surface water	NF and RO	Coagulation and Adsorption (Not available)	Needs a pre-treatment to produce drinking water. NF reduced hardness, COD, TOC, and completely removed microbial content.	[128]
India	Arsenic contaminated water	NF	Nil (Not available)	NF remove arsenic (99.80%) following World Health Organization (WHO) level	[129]
Mozambique	Freshwater	UF	Sand filter of 150 µm and 25µm (Not available)	Permeate flux remained constant and post-chlorination is required at the permeate tank prior to the distribution point to ensure suitable microbiological criteria.	[130]
Brazil	Brackish Water	RO-Desalination	Nil (Not available)	The desalinated water showed rejections ~ 94% for SO_4_^2−^, 97% for TDS and 100% for F^−^.	[131]
Indonesia	Brackish Water	RO-Desalination	Degasifier, coagulation and dual-media filter (Not available)	The groundwater can be treated by RO powered using renewable energy or a simple desalination plant using solar still. Both technologies are efficient and cheap. Modularity allows for upgrades and minimizes operational interruptions when membrane under maintenance.	[132]
Vietnam	Seawater	Air gap membrane distillation (AGMD)	MF HF (46 L/h)	The seawater AGMD desalination proved feasible for both technical and economic. Produce 46 L h^−1^ of high-quality distillate with specific energy consumption of 87 kWh·m^−3^ without any issue of membrane fouling and wetting when dealing with real seawater.	[133]
Vietnam	Wastewater and Seawater	MF, UF, NF, RO, FO and MD	Filtration (Not available)	FO and MD can be used in small-scale systems at low expenses. A membrane offers compactness, system modularization, and lower energy consumption	[134]
Vietnam	Surface Water	NF and ED hybrid process	Electrodialysis (Not available)	ED–NF is an effective alternative for small surface water treatment plants in rural Vietnam. The water quality generated was according VN guideline.	[135]
Southern India	Membrane filtered water and household container water samples	Decentralized membrane filtration	Filtration (Not available)	Membrane filters helped reduce faecal coliform bacteria and decentralized water filtration infrastructure may be effective in places where the microbiological quality of water is not addressed correctly. Initial costs for installation and maintenance are affordable.	[136]
Thailand	Freshwater	Ozonation (Submerged Ceramic MD and UF	AC filter with 50µm (5 m^3^/h)	This multi-stage process ensures efficient drinking water production free from viruses and pathogens. Due to low space requirements, compact treatment units for decentralised units are needed.	[137]
Sri Lanka	Groundwater	Nanofiltration (NF)	Sand and AC filters, cation exchange resin, precision filter (20 m^3^/d)	The NF plant’s permeate water reduce hardness, fluoride, and DOC. Fulfils Sri Lankan drinking water requirements and is well approved by society’s stakeholders.	[138]
China	Reservoir	Hollow fibre UF	Filtration (100,000 m^3^/d)	During the 7-year operation, the UF membrane was effective to avoid breakthrough of organic substance from microorganism metabolic activity.	[70]
China	Raw Water	UF	Coagulation (Not available)	Effective turbidity and other metals removals, including total removal of coliform bacteria. Coagulation process is needed before UF for surface water with high turbidity and varying quality.	[139]
South Africa	Surface Water	Low Pressure UF	Sand Filter (Not available)	UF produce quality potable water at low operating pressures ranging from 100 to 150 kPa hydrostatic pressure. Excellent removal of turbidity and no coliforms or faecal coliforms.	[140]

**Table 3:** General costs comparison between UF membrane and conventional sand/media water treatment systems.

	UF Membrane System	Conventional Sand/Media System
Construction/Capital Cost	Higher	Lower
Operational Cost	Higher	Lower
Maintenance Cost	Higher	Lower
Land Requirement	Lower	Higher

## Data Availability

The datasets used and/or analysed during the current study are available from the corresponding author on reasonable request.
